# Maximum Entropy Exploration in Contextual Bandits with Neural Networks and Energy Based Models

**DOI:** 10.3390/e25020188

**Published:** 2023-01-18

**Authors:** Adam Elwood, Marco Leonardi, Ashraf Mohamed, Alessandro Rozza

**Affiliations:** lastminute.com Group, Vicolo de Calvi, 2, 6830 Chiasso, Switzerland

**Keywords:** machine learning, multi-armed bandit, Thompson Sampling, energy based models

## Abstract

Contextual bandits can solve a huge range of real-world problems. However, current popular algorithms to solve them either rely on linear models or unreliable uncertainty estimation in non-linear models, which are required to deal with the exploration–exploitation trade-off. Inspired by theories of human cognition, we introduce novel techniques that use maximum entropy exploration, relying on neural networks to find optimal policies in settings with both continuous and discrete action spaces. We present two classes of models, one with neural networks as reward estimators, and the other with energy based models, which model the probability of obtaining an optimal reward given an action. We evaluate the performance of these models in static and dynamic contextual bandit simulation environments. We show that both techniques outperform standard baseline algorithms, such as NN HMC, NN Discrete, Upper Confidence Bound, and Thompson Sampling, where energy based models have the best overall performance. This provides practitioners with new techniques that perform well in static and dynamic settings, and are particularly well suited to non-linear scenarios with continuous action spaces.

## 1. Introduction

In recent years, machine learning has been applied to solve a large array of concrete scientific and business problems [1,2,3]. The rapid advancements have mainly been due to the increased access to large datasets and computing resources. However, many real world scenarios require online decision making. They generally do not come with readily available datasets that cover the phase space in question, instead the data must be collected as decisions are made. These kinds of problems generically come under the banner of reinforcement learning, where a sequential series of actions must be made in an environment, where previous decisions influence future decisions.

One class of reinforcement learning problem that is particularly relevant to modern technology businesses is known as the contextual bandit, an extension of the multi-armed bandit problem [4]. In contextual bandit algorithms, actions must be chosen given the state of the system, which is specified by its context. Actions are chosen so as to maximise the total reward over time. The result of a particular action is obtained immediately and can be used to inform future decisions. For optimal performance, these actions should be chosen to trade-off the exploration of phase space with the exploitation of the most rewarding behaviour. Contextual bandits are relevant in many business applications, such as dynamic pricing [5] and recommender systems [6]. Moreover, these approaches have shown great results in the field of advertising and sponsored search actions [7,8,9].

There are many machine learning models capable of making predictions about a reward given an input action and context. Artificial neural networks (NNs) are one of the most popular choices. However, these models are typically brittle, in that they still give confident answers outside of the data distribution they have been trained on, where they are likely to be wrong. A policy for choosing actions in a contextual bandit scenario therefore needs an exploration component added on top of the underlying reward estimator.

One approach to the above issue is to estimate the uncertainties in the predictions made by the neural network. Actions can then be chosen via Thompson sampling, a Bayesian methodology for making sequential decisions under uncertainty [10,11,12]. However, finding accurate and efficient ways of estimating the uncertainties remains challenging.

Another approach is maximum entropy exploration, sometimes known as *Active Inference* or *Boltzmann exploration*. This is also popular in neuroscience as a model of the way the human brain works [13,14,15,16,17,18,19,20]. In maximum entropy exploration, a policy is built that maintains a high entropy over the action space, ensuring that it tries lots of different actions, while still aiming for the best possible reward. This has been introduced for contextual bandit problems with a discrete action space [21]. In this work, we extend this approach to work with a continuous action space.

Energy Based Models (EBMs) are particularly well suited to maximum entropy exploration, due to the close relationship of EBMs with Boltzmann distributions [22]. While straightforward neural networks trained with cross-entropy or mean-squared-error losses can work well as reward estimators, they are prone to brittleness. Conversely, EBMs naturally build uncertainty into their formalisation. Instead of giving a certain answer on the best action to play, energy based functions give a degree of possible actions based on the shape of the energy function. Actions can then be found by sampling from this function with techniques based on Markov Chain Monte Carlo (MCMC). These types of models have been considered in full reinforcement learning scenarios [23,24]. In this work, we introduce a method to apply EBMs based on NNs to contextual bandit problems.

In this paper, we introduce two new contextual bandit algorithms based on maximum entropy exploration. Both algorithms are able to make decisions in continuous action spaces, a key use case that has not been studied as thoroughly as discrete action spaces. Our main contributions can be summarised as follows:

- Introducing a technique for maximum entropy exploration with neural networks estimating rewards in contextual bandits with a continuous action space, sampling using Hamiltonian Monte Carlo;
- A novel algorithm that uses Energy Based Models based on neural networks to solve contextual bandit problems;
- Testing our algorithms in different simulation environments (including with dynamic environments), giving practitioners a guide to which algorithms to use in different scenarios.

## 2. Related Work

As they are very relevant to many industry applications, contextual bandits have been widely studied, with many different algorithms proposed, see for example [4,25,26].

Many of the most successful algorithms rely on linear methods for interpreting the context, where it is easier to evaluate output uncertainty [11,27]. This is necessary, because the most commonly applied exploration strategies, Thompson Sampling [10] and the Upper Confidence Bound (UCB) algorithm [28], rely on keeping track of uncertainties and updating them as data are collected. However, several techniques for non-linear contextual bandit algorithms have been proposed, using methods based on neural networks with different approaches to predicting uncertainties in the output [29,30,31,32].

### 2.1. Entropy Based Exploration in Contextual Bandits

As an alternative to Thompson Sampling and UCB, in this work we focus on entropy based exploration strategies, with an emphasis on their application to non-linear contextual bandit problems. This approach has been researched in the reinforcement learning [33,34] and Multi Armed Bandit literature [19,35].

For the contextual bandit use case, non-linear maximum entropy based exploration with a discrete action space has been considered by [21]. In this case, the non-linearity comes from neural networks, which are used to estimate a reward.

### 2.2. Energy Based Models in Reinforcement Learning

Many problems in machine learning, contextual bandits included, revolve around modelling a probability density function, p(x) for $x\in R^{D}$. These probability densities can always be expressed in the form of a scalar energy function $E_{\theta }\left(x\right)$ [36]:

(1) $$p\left(x\right)=\frac{\exp(-E_{\theta }\left(x\right))}{\int_{x\prime }\exp\left(-E_{\theta }\left(x\prime \right)\right)dx\prime },$$

which allows many machine learning problems to be reformulated as an energy based modelling task [37]. The difficulty with this reformulation comes in estimating the integral in the denominator of Equation (1), which is usually intractable. However, if this difficulty can be overcome, a scalar function, $E_{\theta }\left(x\right)$, is learned, which can be evaluated at any value of x, providing a fully generative model.

Another advantage of EBMs in a reinforcement learning setting is that sampling from them naturally leads to maximum entropy exploration [22]. This has been applied to solve full reinforcement learning problems in both model-based [24] and model-free [23,38] formulations. However, it has not yet been applied to specifically solve the contextual bandit problem.

## 3. Algorithms to Solve Contextual Bandit Problems with Maximum Entropy Exploration

In this section, we introduce two classes of algorithms for solving contextual bandit problems with NNs, using exploration strategies based on entropy maximisation. In each case, the algorithm defines a policy, $\pi (a|s_{i})$, which gives the probability of playing action *a*, given the observation of state $s_{i}$. The policy is applied by sampling actions from the policy, *a*∼π, at a particular time step. This policy is then updated given the rewards observed in previous time steps by retraining the NNs.

### 3.1. Contextual Bandit Problem Formulation

Contextual bandit problems require an algorithm to make the choice of an action, a∈A (where A is an action space), upon observing the context state of the environment, s∈S (where S is a context space). Upon making the action, a reward, r∈R, is received. For each state observed, an action is chosen and the reward recorded. This results in a dataset, X, being built up over a run, consisting of triplets, $\{s_{i},a_{i},r_{i}\}\in X$, where i∈N is the time step of a particular triplet. At any step *i*, the data available for choosing the action $a_{i}$ consist of the set of triplets $\left\{s_{j},a_{j},r_{j}\right\}$, where j<i.

The goal of the problem is to maximise the expected reward over an indefinite time horizon, where an arbitrary number of actions, *N*, can be played. This is usually measured in terms of the regret:

(2) $$R_{N}=\sum_{i=0}^{N}\left[r_{i}^{*}-r_{i}^{a_{i}}\right],$$

where $r_{i}^{*}$ is the best possible reward at the time step *i* and $r_{i}^{a_{i}}$ is the reward at time step *i* received by the action played, $a_{i}$. A more successful action choosing policy will have a lower regret.

In this work, we assume A∈R; $S\in R^{n}$, where *n* is the dimension of the context vector and depends on the particular problem being considered; and R∈R, where many of the problems considered assume R∈[0,1].

### 3.2. Maximum Entropy Exploration

First, we define a reward estimator, ${\hat{r}}_{\theta }\left(s_{i},a\right)$, which gives the expected reward of action *a* in state $s_{i}$ and is parameterised by the vector θ, similar to the approach in [21]. In maximum entropy exploration, the policy is defined as follows:

(3) $$\pi \left(a|s_{i}\right)=\underset{\pi }{arg max}\left(E_{a∼\pi }\left[{\hat{r}}_{\theta }\left(s_{i},a\right)\right]+\alpha H\left(\pi \right)\right),$$

where $H\left(\pi \right)=E_{a∼\pi }\left[-\log\left(\pi \right)\right]$ is the Shannon entropy. This can then be solved with a softmax:

(4) $$\pi \left(a|s_{i}\right)=\frac{e^{{\hat{r}}_{\theta }\left(a,s_{i}\right)/\alpha }}{\int_{a^{\prime }}e^{{\hat{r}}_{\theta }\left(a^{\prime },s_{i}\right)/\alpha }da^{\prime }}.$$

This approach finds a policy that trades off maximising the expected reward with a chosen action (the first term in Equation (3)) with trying a range of different actions, which give a large Shannon entropy (the second term in Equation (3)). The degree of this trade off is controlled by the $\alpha \in R^{+}$ parameter, which is typically chosen to be at the same scale as the expected reward. Larger α values result in more exploration. Models for ${\hat{r}}_{\theta }$ should be chosen to have a fairly flat prior across the states and actions upon initialisation, which will encourage exploration in the early stages of a contextual bandit run. As time progresses and ${\hat{r}}_{\theta }$ becomes more certain, the entropy term ensures exploration is not reduced too prematurely. In the case of static environments, it is also desirable to reduce the α value over time, ensuring the total regret of the algorithm is bounded as N→∞ [21,39].

### 3.3. Maximum Entropy Exploration with Neural Networks Modelling Reward

We build our reward estimator, ${\hat{r}}_{\theta }\left(a,s_{i}\right)$, with a neural network trained to predict the reward, $r_{i}$. With this methodology, we model the expected reward given a certain action. This then allows us to select an action based on this expectation value. As there is no explicit model of the environment, this can be thought of as analogous to the suite of “model free” techniques in reinforcement learning [40].

In the general case, the neural network can be treated as a regressor with a loss based on the mean-squared-error. In the binary reward case (R∈[0,1]), the network can be treated as a classifier and trained with the binary cross-entropy. Given the reward estimator, samples are drawn from π to choose actions online, and ${\hat{r}}_{\theta }$ is refit as we collect more data. However, due to the fact that the integral in the denominator of Equation (4) is likely intractable, sampling is not always trivial. We therefore take two different approaches to approximate the integral.

#### 3.3.1. Discrete Action Sampling

In the case where there is a predefined discrete set of actions, the integral in Equation (4) can be rewritten as a sum over all possible actions, $a^{\prime }$, and explicitly calculated:

(5) $$\pi \left(a|s_{i}\right)=\frac{e^{{\hat{r}}_{\theta }\left(a,s_{i}\right)/\alpha }}{\sum_{a^{\prime }}e^{{\hat{r}}_{\theta }\left(a^{\prime },s_{i}\right)/\alpha }}.$$

This has the advantage of being easy to implement and applies to a wide range of contextual bandit problems. However, it has the limitation that the time for calculating π scales linearly with the number of actions to be sampled, so is not easily applicable to problems with large or continuous action spaces.

#### 3.3.2. Continuous Action Sampling

Equation (5) has the form of a posterior probability distribution, which is one of the quantities in Bayesian statistics, so techniques for sampling from this distribution are widely covered in the literature. To draw samples from $\pi (a|s_{i})$ in a continuous action space, we can make use of MCMC sampling algorithms. In our case, we employ the Hamiltonian Monte Carlo (HMC) algorithm [41,42], due to its wide usage and availability of suitable implementations [43,44].

This solution works in the general case of a continuous action space, where a∈R. However, in many cases, the action space is constrained such that any particular action *a* is subject to $a\in [a^{lower},a^{upper}]$, where $a^{lower}$ and $a^{upper}$ denote the upper and lower bounds of possible actions. To deal with this constraint, we can modify the reward estimator to include the constraints, $r_{\theta }^{c}$, to return a large negative number when the action is outside the bounds:

(6) $$\begin{array}{cc} {\hat{r}}_{\theta }^{c}\left(a,s_{i}\right) & ={\hat{r}}_{\theta }\left(a,s_{i}\right)\;\mathrm{when}\;a\in \left[a^{lower},a^{upper}\right]\\ {\hat{r}}_{\theta }^{c}\left(a,s_{i}\right) & =-\infty \;\;\;\;\;\;\;\;\mathrm{otherwise}. \end{array}$$

Replacing ${\hat{r}}_{\theta }$ with ${\hat{r}}_{\theta }^{c}$ in Equation (5) when carrying out the HMC sampling will then ensure the actions that are sampled are within the constraints.

A summary of the algorithms described in this section can be seen in Algorithm 1, where the sampling procedure will change depending on whether the action space is discrete or continuous, as described above. At every timestamp *i*, the algorithm parameterized by θ has to choose an action $a_{i}$ upon the given context $s_{i}$. Then it receives a reward $r_{i}$ and the dataset X is updated with the triplet $\left\{s_{i},a_{i},r_{i}\right\}$. Every *k* iteration, the model is trained over the dataset X. In the following, these algorithms will be named *NN Discrete* or *NN HMC*, respectively.
**Algorithm 1** Maximum entropy exploration with neural networksInput: $\alpha ,N,\theta_{0},X_{0},k$**for** i=1,…,N **do**    Receive context $s_{i}$ and choose $a_{i}∼\pi_{i}$ where    $\pi_{i}\left(a|s_{i}\right)=\frac{e^{{\hat{r}}_{\theta_{i-1}}\left(a,s_{i}\right)/\alpha }}{\int_{a^{\prime }}e^{{\hat{r}}_{\theta_{i-1}}\left(a^{\prime },s_{i}\right)/\alpha }da^{\prime }}$    Agent receives reward $r_{i}$    Add the triplet $\left\{s_{i},a_{i},r_{i}\right\}$ to the dataset X    Every *k* steps train the model ${\hat{r}}_{\theta }$:    $\theta_{i}={arg min}_{\theta }\sum_{\{s_{j},a_{j},r_{j}\}\in X}\left|r_{j}-{\hat{r}}_{\theta }\left(a_{j},s_{j}\right)\right|$**end for**

### 3.4. Maximum Entropy Exploration with Energy Based Models (EBMs)

Energy-based models allow us to model the probability of choosing an action given a reward, $p(a|s_{i},r)$, with a scalar-valued energy function $E_{\theta }\left(a,s_{i},r\right)$, parameterised by θ, which is then marginalised over the state and reward spaces:

(7) $$p\left(a|s_{i},r\right)=\frac{\exp(-E_{\theta }\left(a,s_{i},r\right))}{\int_{a^{\prime }}\exp\left(-E_{\theta }\left(a^{\prime },s_{i},r\right)\right)da^{\prime }}.$$

This then allows us to find the optimal policy by finding the probability of an action to obtain an optimal reward, $r^{*}$:

(8) $$\pi \left(a|s_{i}\right)=\frac{\exp(-E_{\theta }\left(a,s_{i},r^{*}\right)/\alpha )}{\int_{a^{\prime }}\exp\left(-E_{\theta }\left(a^{\prime },s_{i},r^{*}\right)/\alpha \right)da^{\prime }}.$$

Drawing samples of actions from this distribution with MCMC techniques will naturally carry out a maximum entropy exploration policy, where the degree of exploration can again be controlled by the size of α [22].

This methodology is particularly well suited to the case of binary rewards, R∈[0,1], as it is easy to choose the optimal reward: $r^{*}=1$.

Contrary to the previous algorithm, when solving the contextual bandit problem with EBMs, we model the probability of an action acting on the environment so as to obtain a certain reward. This is analogous to modelling the probability of a state transition on the environment, so is more in line with the “model based” techniques discussed in the reinforcement learning literature [24,45,46].

#### 3.4.1. Training EBMs to Solve Contextual Bandit Problems

A generic energy function can be learned by minimising $E_{\theta }\left(a,s,r\right)$ for the most probable {s,a,r} triplets and maximising it for the least probable triplets that currently have a low energy [36]. A simple form of loss function that achieves this goal is [47,48]:

(9) $$L=E_{x^{+}∼p_{D}}\left(E_{\theta }\left(x^{+}\right)\right)-E_{x^{-}∼p_{\theta }}\left(E_{\theta }\left(x^{-}\right)\right),$$

where $x^{+}$ represent {s,a,r} triplets drawn from the historical dataset, X, while $x^{-}$ represent triplets sampled from the model.

This approach works in the general case and has the advantage of learning a generative model, which can be used to find any conditional probability distribution. However, training in this way is intensive and unstable, as it requires MCMC sampling when evaluating the loss function and a large existing dataset.

In the contextual bandit use case, however, we are only interested in learning $\pi (a|s_{i})$ for the optimal reward, so we can simplify this approach by reducing the input dimensions to the energy function and only learning energies for the optimal rewards:

(10) $$E_{\theta }\left(a,s_{i}\right)\equiv E_{\theta }\left(a,s_{i},r^{*}\right).$$

This approach is both easier to train and requires fewer initial training examples.

After experimenting with the different forms for L described in [36], we settled on a logarithmic form, which had the most consistent stable performance:

(11) $$L=\log(1+\exp\left(E_{\theta }\left(a^{+},s\right)-E_{\theta }\left(a^{-},s\right)\right)),$$

where $a^{+}$ are actions that result in an optimal reward, $r^{*}$, and $a^{-}$ are actions that result in a suboptimal reward (0 in the binary case). These values and their corresponding states, $s_{i}$, are taken from historical data.

#### 3.4.2. Architectures for EBMs

To be able to train EBMs, it is convenient to choose an architecture that can easily be updated with stochastic gradient descent, while avoiding instabilities in the training. Such instabilities include arbitrarily large or small energy values and energy collapse, where a model learns a minimal value of the energy function across all input values. These criteria can be fulfilled by combining two neural networks, $f_{\phi }$ and $g_{\psi }$, in an *implicit regression* architecture [36], where:

(12) $$\begin{array}{c} f_{\phi }:a\to R\\ g_{\psi }:s_{i}\to R. \end{array}$$

**Figure 1:**
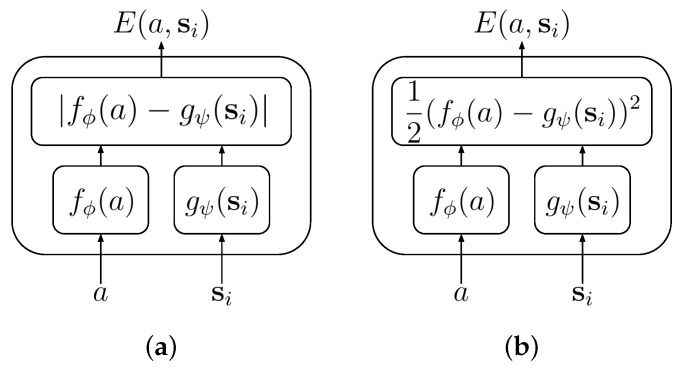
Example of the implicit regression architecture: (**a**): linear case, (**b**) quadratic case.

The energy function can then be defined in two different ways, either linear:

(13) $$E\left(a,s_{i}\right)=\left|f_{\phi }\left(a\right)-g_{\psi }\left(s_{i}\right)\right|$$

or quadratic:

(14) $$E\left(a,s_{i}\right)=\frac{1}{2}{\left(f_{\phi }\left(a\right)-g_{\psi }\left(s_{i}\right)\right)}^{2},$$

as depicted in the Figure 1. In both these cases, the energy function is bounded from below by 0 and requires two independent networks to both learn the same value for all inputs to result in energy collapse. Both of these features combine to help improve training stability. In the following, we consider the quadratic combination.

The added advantage of using neural networks in the architecture is that it allows us to easily draw MCMC samples from π using Stochastic Gradient Langevin Dynamics (SGLD), as presented by [48,49,50]. This algorithm works by starting from a random point, ${\tilde{x}}^{0}$, and iterating in the direction of higher probability with the gradients of the energy function [51]. Noise, ω∼N(0,σ), is added to each gradient step to ensure that the sampling fully captures the underlying probability distribution. This chain is carried out for *K* steps, where the *k*-th step, ${\tilde{x}}^{k}$, is calculated as follows:

(15) $${\tilde{x}}^{k}\leftarrow {\tilde{x}}^{k-1}-\eta \nabla_{x}E_{\theta }\left({\tilde{x}}^{k-1}\right)+\omega ,$$

where η is the sample gradient step size.

The full procedure required to solve the contextual bandit problem with energy based models is summarised in Algorithm 2: At every timestamp *i*, the algorithm parameterized by θ has to choose an action $a_{i}$ upon the given context $s_{i}$. Then, it receives a reward $r_{i}$ and the dataset X is updated with the triplet $\left\{s_{i},a_{i},r_{i}\right\}$. Every *c* iteration, the model is trained over the dataset X. In the rest of this work, this algorithm will be referred to as the *EBM* algorithm.
**Algorithm 2** Contextual bandit with Energy Based ModelsInput: $N,\theta_{0},X_{0},K,c,\alpha ,a_{max},a_{min},\eta ,\sigma$**for** i=1,…,N **do**    Choose $a_{i}∼\pi_{i}$ with SGLD, ${\tilde{a}}^{0}∼U\left(a_{min},a_{max}\right)$    **for** k=1,…,K **do**        Draw sample for noise ω∼N(0,σ)        ${\tilde{a}}^{k}\leftarrow {\tilde{a}}^{k-1}-\eta \nabla_{x}E_{\theta_{i-1}}\left({\tilde{a}}^{k-1},s_{i}\right)/\alpha +\omega$    **end for**    Play action ${\tilde{a}}^{K}$, receive $r_{i}$, update X    Every *c* steps train $E_{\theta }$ in batches:    $\theta_{i}={arg min}_{\theta }\sum_{X}\log\left(1+e^{E_{\theta }\left(a^{+},s_{j}\right)-E_{\theta }\left(a^{-},s_{j}\right)}\right)$**end for**

#### 3.4.3. Evolution of the Energy Distribution with Dataset Size

One key property of any model for solving contextual bandit problems is that its uncertainty about the correct action to play decreases as more relevant data are collected. This ensures convergence on the best strategy, which gradually reduces the exploration over time as the space of plausible actions decreases.

**Figure 2:**
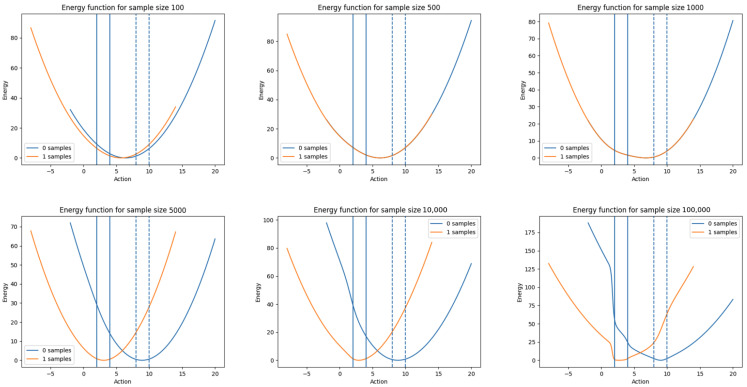
The evolution of an energy function as the training sample size is increased from 100 to 100,000 in an environment with two distinct context categories (labeled 0, blue, and 1, orange) with optimal actions around 3 and 9, respectively.

For an energy based model, this is visible as the energy function decreasing in width around the optimal action ranges as the number of samples used for training increases. With the model described in this section, we have empirically justified that we obtain this desired behaviour. An example of this can be seen in Figure 2, where the evolution of the energy function is plotted as the number of samples is increased for a training set with two contexts, which both have distinct optimal action ranges. It can be seen that, as the number of samples increases, the model learns to distinguish the two different contexts and narrows in on the optimal action range. Any appropriate sampling approach will therefore sample widely initially and then converge onto the optimal action for each context.

## 4. Experiments

To test the algorithms presented so far, we carried out a series of experiments in different contextual bandit simulation environments. We considered both static environments, where the optimal action given a context does not vary over time, and dynamic environments, where it does. This is of particular relevance to algorithms that will be deployed in real-world environments, which are almost never static. This fact also motivated us to focus on settings with continuous action spaces, which are particularly relevant to many industrial use cases. We focused on experiments in a simulation environment due to the lack of relevant benchmarks with dynamic and continuous action spaces in the literature with real datasets.

### 4.1. The Simulation Environments

Our simulation environment requires a model to play a series of actions, so as to maximise its total expected reward. Any particular reward is obtained immediately after an action. Let *N* be the number of rounds, $s\in R^{h}$ the context vector that the policy observes, and $r_{i}\in \left[0,1\right]$ the the reward given by playing the action $a_{i}\in R$ on round *i* given the context $s_{i}$.

Let *J* be the number different reward functions $\rho_{j}:a\to \left[0,1\right]$. Each context, s, belongs to a particular reward function, $\rho_{j}$.

The reward function $\rho_{j}$ is modelled by the probability density function of a Gaussian distribution $N(\mu_{j},\sigma_{j}^{2})$ parameterized by $\mu_{j}$ and $\sigma_{j}^{2}$, where $\mu_{j}$ indicates the optimal action that has to be played for that particular reward function. Given an action, *a*, the reward function first computes the expected probability of having a reward of 1 by taking the value of the Gaussian at *a*, $P_{j}\left(r=1|a\right)$. It then draws a sample from a uniform distribution between 0 and 1, $u_{a}∼U\left(0,1\right)$ and uses it to calculate the reward:

(16) $$\rho_{j}\left(a\right)=\left\{\begin{array}{cc} 1 & \mathrm{if}\;u_{a}<P_{j}\left(r=1|a\right)\\ 0 & \mathrm{otherwise} \end{array}\right.$$

**Figure 3:**
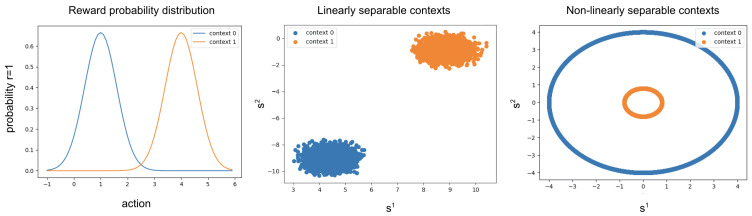
Example of a simulation environment used for testing the algorithms with two contexts. The probability of receiving a reward given an action depends on the context as shown on the left. The linearly and non-linearly separable contexts are shown in the centre and on the right, respectively.

An example of reward functions for a two-context environment can be seen on the left of Figure 3.

To make the environment dynamic, it is possible to only modify $\mu_{j}$ based on the current round of the simulation *i*, making it a function of the timestep, $\mu_{j}\left(i\right)$.

### 4.2. Experimental Setup

We designed our synthetic environment to have multiple homogeneous contexts, each of which is associated to a reward function.

To test the capabilities of each proposed method, contexts s can be either linearly or non-linearly separable. For the linear case, we generated multiple isotropic Gaussian blobs in a three-dimensional space, h=3. Each blob was generated from a Gaussian with a fixed standard deviation of 0.4 and a random mean. In the non-linearly separable case, two different set of contexts were generated in a two-dimensional space, h=2: a large circle containing a smaller one. Both the circles were zero centered, and had a radius of 4.0 and 0.8, respectively. Examples of similar contexts can be seen on the right and in the centre of Figure 3.

The experiments consisted of N=10,000 observations with J=2 different reward functions. They both had the same variance, 0.6. In the static environments setting, the mean value, $\mu_{j}$, was set to 1 and 4, respectively, while in the dynamic setting, it was perturbed by a cosine function:

(17) $$\mu_{j}\left(i\right)=\mu_{j}+\cos\left(\frac{i}{500}\right)+0.5.$$

### 4.3. Baseline Algorithms

We considered the most popular bandit algorithms as baselines against which we compared our algorithms. As a baseline that does not take into account the context, we looked at two Multi–Armed Bandit (MAB) approaches, namely the Upper Confidence Bound bandit algorithm (UCB1) [28] and Thompson Sampling (TS) [10]. These algorithms have an arm for each action and learn the best action to play on average. As a baseline that takes into account the context, we chose the linear UCB algorithm (linUCB) [27] and the linear TS algorithm (linTS) [11].

### 4.4. Specific Configurations of the Algorithms

Before running all the experiments, all the parameters of the methods were manually fine tuned to achieve the best results.

For the discrete action algorithms (including the MAB and linUCB algorithms), the possible actions were chosen to be between 0.2 and 5.2 with an offset of 1, giving six possible actions. None of the possible discrete actions were set on the optimal action point for the simulation environment to avoid giving the discrete algorithms an unfair advantage with respect to the continuous algorithms.

For linUCB, the α parameter was set to 0.05, which was chosen from a hyperparameter tuning of values between 0 and 10, while for linTS the *v* parameter was fixed at 1 for the whole simulation.

One difficulty with the neural network policies is that they run into the cold-start problem, where the they are unable to make any decisions before they have been trained on some data. For these methods, we set up a warm-up phase in which the policy randomly explores the action space by sampling the actions from a uniform distribution. After this initial phase, the algorithm should have enough data to train. In the experiments, we set a warm-up time of 1000 steps, with actions sampled from a uniform distribution *a*∼U(0.5,5.5).

Both the *NN Discrete* and the *NN HMC* algorithms share the same neural network architecture, which is a simple multilayer perceptron (MLP) with two hidden layers of 50 neurons with ReLu activation functions and a single neuron with a sigmoid activation function on the output. They were trained after every 100 steps for 10 epochs with a batch size of 2. The alpha entropy exploration term was set to 0.1 and 0.5 for *NN discrete* and the *NN HMC*, respectively.

For the *NN HMC* algorithm, the initial state of the MCMC was set to 2.5, with a step size of 1 and 100 burn in steps. Adam [52] was used as an optimiser over the binary cross-entropy loss with a learning rate of 0.001.

For the EBM algorithm, both $f_{\phi }$ and $g_{\psi }$ share the same architecture with sightly different parameters. They are both MLPs composed of four layers, each of which have sigmoid activation functions, except the last block which is just a linear output. Sigmoids were chosen as they were shown empirically to perform better than ReLu activation functions.

The first two blocks of $f_{\phi }$ have an output size of 256, while the following two have output sizes of 128 and 1, respectively. Instead, $g_{\psi }$ is composed of internal layers with an output size of 128.

The EBM was trained every 100 environment steps for a total of 150 epochs, with a learning rate of 0.005, a dropout of 0.2 on every internal layer, and a batch size of 128. For each training iteration after the first, the weights of the MLPs were initialised as the resulting weights of the previous training run. For the action sampling, the exploration term α was set to 10, the gradient step size η was set to 0.2, ω was sampled from a Gaussian with σ=0.005, and 100 SGLD steps were carried out to choose the action.

To deal with instabilities in the training of the EBMs, for the first training iteration we initialised the weights randomly and retrained the model from 10 to 55 times, searching for the model that minimises the difference between a played action and the action that brings a positive reward. Looking at the actions in the warm-up phase that resulted in positive rewards, we evaluated the actions that the model would play, by drawing a single sample from π. We computed the average absolute difference over all of these samples and took the model that minimises this average, or stopped if we found a model with an average difference of less than 0.6.

### 4.5. Results

In Table 1, we report the results of all the considered algorithms over all the aforementioned environments: linearly separable (Linear) and non-linearly separable (Circle), both static and dynamic. In each case, the regret is calculated as shown in Equation (2), where the best possible reward is obtained from the simulation environment. Each run was carried out five times, with the mean and standard deviation of stochastic regret reported in the table.

Across most of the experiments, the EBM algorithm performed the best, showing a good ability to adapt to both linear and non-linear contexts, along with some adaptability to dynamic environments. The main difficulties with the EBM algorithm came from instabilities in training, which could occasionally lead to a bad performance. This resulted in the large standard deviation in the regret for the static Circle environment.

The *NN Discrete* algorithm also performed well, especially with non-linear contexts, but had less flexibility to deal with dynamic environments. In these cases, the ability to carry out continuous action sampling brought an advantage to *NN HMC*. However, the continuous action sampling was less competitive in static environments. This could likely be improved by further tuning the sample step size, reducing it in environments with less variability.

The UCB1 and TS algorithms, which do not take the context into account, were not able to compete with the algorithms that did. However, they did provide a useful baseline for tuning the other algorithms. The linUCB and the linTS algorithms, which do take the contexts into account, were competitive in the linearly separable environments, but could not deal with the non-linearly separable environment.

## 5. Conclusions

We have introduced algorithms to solve contextual bandits in both continuous and discrete action spaces, making use of maximum entropy exploration. These algorithms are based on neural networks and work either by estimating the reward given a particular action and context, or by modelling the best action probability with an energy function.

Overall, the EBM algorithm performed best in a series of simulation experiments, showing good potential for applications to contextual bandit problems with continuous action spaces. In discrete action spaces, the *NN Discrete* algorithm also performed comparably well in the non-linear case and suffered from fewer training instabilities.

In future work, it would be useful to research other techniques to reduce the instability of training the EBM as in [53]. It would also be worth investigating more intelligent cold-start policies to improve algorithm performances in the initial steps such as linUCB, linTS or even *NN Discrete*.

## Figures and Tables

**Table 1:** Average stochastic regret and best stochastic regret of the considered algorithms over five runs for different environments: non-linearly separable (Circle) and linearly separable (Linear), both static and dynamic. The highest results are highlighted in **bold** while the second highest results are marked in *italic*.

	LINEAR				CIRCLE			
	STATIC		DYNAMIC		STATIC		DYNAMIC	
**Experiment**	Average Stochastic Regret	Best Stocastic Regret	Average Stochastic Regret	Best Stocastic Regret	Average Stochastic Regret	Best Stocastic Regret	Average Stochastic Regret	Best Stocastic Regret
EBM	**551 ± 123**	**450**	**3275 ± 59**	*3194*	**987 ± 402**	**613**	**3307 ± 71**	**3208**
NN HMC	2229 ± 100	2080	3578 ± 88	3502	2074 ± 254	1642	*3572 ± 42*	*3532*
NN Discrete	1166 ± 98	1046	3720 ± 75	3627	*991 ± 66*	*879*	3645 ± 37	3616
UCB1	3721 ± 66	3675	4508 ± 127	4319	3683 ± 68	3608	4002 ± 63	3900
TS	3552 ± 87	3480	4868 ± 65	4808	3619 ± 24	3583	4760 ± 65	4656
linUCB	1196 ± 632	633	3822 ± 497	**3134**	4446 ± 558	3955	4933 ± 66	4842
linTS	*558 ± 47*	*481*	*3447 ± 50*	3375	4473 ± 56	4413	5012 ± 53	4952

## Data Availability

The data presented in this study are openly available in GitHub at https://github.com/aelwood/contextual-bandit-playground.
